# BDNF-dependent consolidation of fear memories in the perirhinal cortex

**DOI:** 10.3389/fnbeh.2013.00205

**Published:** 2013-12-17

**Authors:** Brigitte Schulz-Klaus, Volkmar Lessmann, Thomas Endres

**Affiliations:** ^1^Tierphysiologie, Institut für Neurobiologie, Universität TübingenTübingen, Germany; ^2^Medizinische Fakultät, Institut für Physiologie, Otto-von-Guericke Universität MagdeburgMagdeburg, Germany; ^3^Center for Behavioral Brain Research (CBBS), Otto-von-Guericke Universität MagdeburgMagdeburg, Germany

**Keywords:** perirhinal cortex, brain-derived neurotrophic factor, fear potentiated startle, fear learning, consolidation

## Abstract

In the recent years the perirhinal cortex (PRh) has been identified as a crucial brain area in fear learning. Since the neurotrophin brain-derived neurotrophic factor (BDNF) is an important mediator of synaptic plasticity and also crucially involved in memory consolidation of several learning paradigms, we analyzed now whether fear conditioning influences the expression of BDNF protein in the PRh. Here we observed a specific increase of BDNF protein 120 min after fear conditioning training. In order to test whether this increase of BDNF protein level is also required for the consolidation of the fear memory, we locally applied the Trk receptor inhibitor k252a into the PRh during this time window in a second series of experiments. By interfering with Trk-signaling during this critical time window, the formation of a long-term fear memory was completely blocked, indicated by a complete lack of fear potentiated startle 1 day later. In conclusion the present study further emphasizes the important role of the PRh in cued fear learning and identified BDNF as an important mediator for fear memory consolidation in the PRh.

## Introduction

Fear learning is a very efficient and evolutionarily highly beneficial learning mechanism that increases the survival probability of an individual in dangerous situations (e.g., Maren, 2001; Pape and Pare, 2010). On the other hand dysfunctions in fear learning are supposed to be one of the major causes for the development of several anxiety disorders, like PTSD (see e.g., Koenigs and Grafman, 2009; Jovanovic and Ressler, 2010; Mahan and Ressler, 2012). Thus the understanding of the underlying neuronal mechanisms and circuitries might lead to novel treatment strategies for several anxiety disorders.

The key brain area mediating cued fear learning in higher vertebrates is the amygdala complex (Maren, 2001; Fanselow and Gale, 2003; LeDoux, 2007; Pape and Pare, 2010). Chronic and temporary lesions of the lateral/basolateral complex of the amygdala block the acquisition, consolidation and expression of fear (e.g., Johansen et al., 2011; Sierra-Mercado et al., 2011). In addition, fear learning alters synaptic plasticity in this area (e.g., Rogan et al., 1997; Sigurdsson et al., 2007). Besides the amygdala, other brain areas have also been identified to be crucially involved in cued fear learning. Among these areas are the prelimbic ventromedial prefrontal cortex (PL; e.g., Burgos-Robles et al., 2009; Choi et al., 2010; Sierra-Mercado et al., 2011) and the perirhinal cortex (PRh; e.g., Schulz et al., 2004; Furtak et al., 2007; Kealy and Commins, 2011; Kent and Brown, 2012). Interestingly, the PRh has strong reciprocal projections to many brain areas that are linked to emotional learning and fear conditioning in particular, like the amygdala (e.g., Pikkarainen and Pitkanen, 2001), the medioprefrontal cortex (e.g., Heidbreder and Groenewegen, 2003), the thalamus (e.g., McIntyre et al., 1996) and the hippocampus (e.g., Kloosterman et al., 2003), thus making the PRh an important part in the neuronal circuitry mediating the formation of fear memories. However, the concrete involvement of PRh in fear learning as well as the underlying neuronal processes is still not fully understood (compare e.g., Kealy and Commins, 2011).

The neurotrophin brain-derived neurotrophic factor (BDNF) is an important mediator for synaptic plasticity and memory formation, acting via activation of TrkB (tropomyosin-related kinase B) receptors (Bekinschtein et al., 2008; Gottmann et al., 2009; Cowansage et al., 2010). Several recent studies demonstrated also an important role of BDNF in synaptic plasticity in the amygdala (i.e., long-term potentiation: Musumeci et al., 2009; Meis et al., 2012) as well as for cued fear learning (Rattiner et al., 2004a,b, 2005; Ou and Gean, 2006; Jones et al., 2007; Ou et al., 2010; Andero et al., 2011; Endres and Lessmann, 2012; Psotta et al., 2013). In addition, for proper consolidation of fear memories BDNF-TrkB-signaling is also required in the PL (Choi et al., 2010). In the PRh, BDNF has so far been shown to be important for object recognition memory (Hopkins and Bucci, 2010; Munoz et al., 2010; Hopkins et al., 2011; Callaghan and Kelly, 2012). Since the PRh is supposed to be involved in the consolidation of both contextual and cued fear memories (Suzuki, 1996; Sacchetti et al., 1999; Burwell et al., 2004), it seems reasonable to assume that BDNF signaling in the PRh might be involved in the consolidation processes of fear memories. However, this issue has not been analyzed so far. Therefore, we analyzed in the present study, whether BDNF in the PRh is required for cued fear learning. In order to test this, we first applied a fear conditioning paradigm that has been shown to rely on PRh function (Schulz et al., 2004) and looked for the expression of BDNF-protein in the PRh upon fear learning. Therefore, we analyzed the amount of BDNF protein in the PRh at distinct time points after fear conditioning training by using a sensitive BDNF-ELISA. Here we observed a strong increase in BDNF protein 120 min after fear conditioning training. In a second series of experiments, we locally injected the unspecific tyrosine kinase inhibitor k252a at the time point matching the peak of BDNF protein expression in the PRh to inhibit TrkB kinases. The fear memory of the animals was assessed by applying the fear potentiated startle paradigm (Davis et al., 1993; Fendt and Fanselow, 1999; Fendt and Koch, 2013). Here we observed complete inhibition of the formation of fear memories, indicated by the absence of fear potentiated startle. In conclusion these results suggest that BDNF signaling in the PRh is required for the successful consolidation of fear memories. However, the involvement of other tyrosine kinases also affected by k252a (i.e., TrkA, TrkC) cannot be excluded.

## Materials and Methods

### Animals

For the present study 56 male adult (220–320 g) Sprague-Dawley rats (Charles River, Sulzfeld, Germany) were used. They were housed in groups of 4–6 animals under a 12 h/12 h light dark cycle (lights on at 7:00 a.m.) and had free accesses to food and water. All experiments were performed in accordance with the ethical guidelines for care and usage of laboratory animals and were approved by the local animal care committee (Regierungspräsidium Tübingen: ZP 3/06).

### Surgery

Rats were deeply anesthetized with ketamine/xylazine (9:1, 100 mg/kg bodyweight) and fixed into a stereotaxic device. Under stereotaxic control two cannulas (0.7 mm in diameter) were implanted bilaterally into the brain, aiming at the PRh (+2.6 mm caudal, ±6.2 mm lateral and 7.6 mm ventral in relation to Bregma (coordinates according to Paxinos and Watson, 1997)). The cannulas were fixed with dental cement and three anchoring screws on the skull bone. After surgery the rats were allowed at least 7 days to fully recover before the behavioral testing started.

### Fear conditioning

For fear conditioning two identical dark boxes (38 × 60 × 28 cm^3^) were used. A 15 W white light served as the conditioned stimulus (CS) that was paired with a 0.5 s lasting 0.6 mA foot shock, which served as unconditioned stimulus (US). Initially the animals received 5 min to adapt to the conditioning chamber and then received 10 CS-US pairings with variable inter-trial intervals (1–3 min). The US presentation (0.5 s) always co-terminated with the light presentation (3.7 s). As control group for the BDNF-protein quantification, a group of animals was pseudo conditioned by receiving the same number of CS und US presentations, but in an unpaired manner.

### Intracranial injections

**Figure 1:**
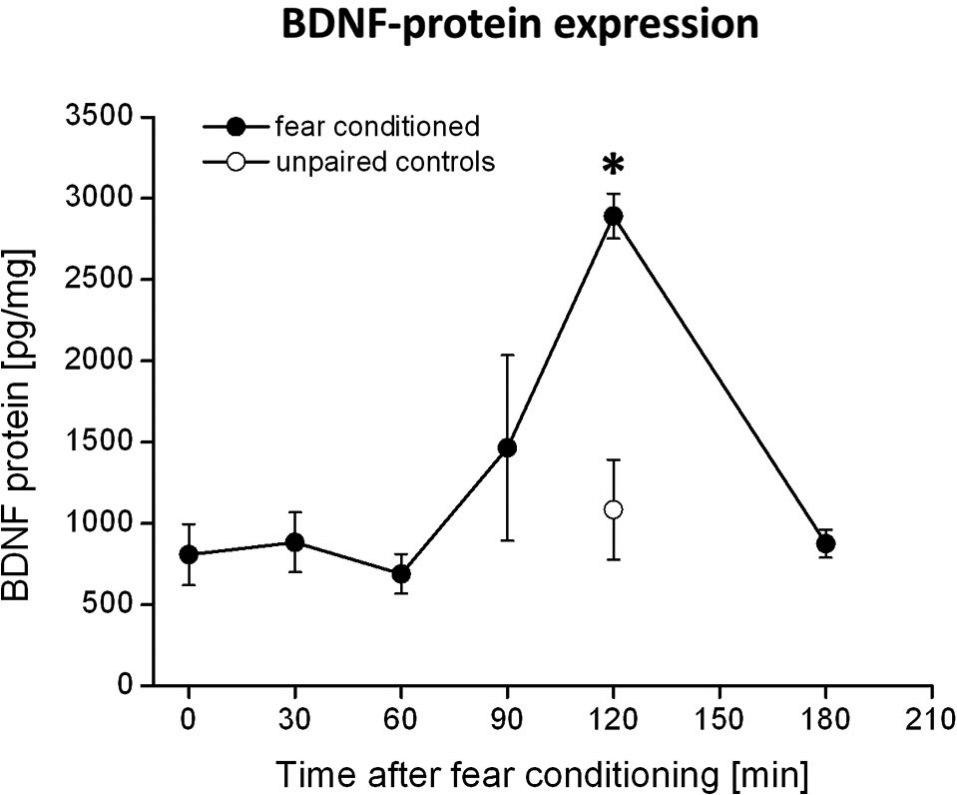
**BDNF protein expression in the PRh after fear conditioning training**. Animals were fear conditioned, sacrificed at distinct time points after fear conditioning and the amounts of BDNF in the PRh were quantified by ELISA. We observed a strong increase in BDNF protein 120 min after fear conditioning training (solid symbols). This increase was absent in the control group (open symbols) which received the CS and US in an unpaired manner. * Represents a *p*-value < 0.05.

The rats received microinjections of either k252a (150 μM, 0.3 μl per side, Alomone labs, Israel) dissolved in DMSO (Sigma-Aldrich, Germany) or DMSO as control via injection cannulas (0.4 mm in diameter) directly into the PRh. The drugs were infused bilaterally with a velocity of 0.1 μl/10 s. The cannulas were left in place for additional 2 min in order to allow diffusion into the surrounding tissue. Since k252a is supposed to require approximately 30 min to attain its full inhibitory effect on Trk receptors (compare e.g., Ou et al., 2010), the drugs were infused 90 min after the fear conditioning training has ended, to block Trk activity at 120 min when we observed the maximum increase in BDNF protein in the PRh (compare Figure 1).

### Fear potentiated startle

The setup to measure the startle amplitudes was located in a sound attenuating test chamber (100 × 80 × 60 cm^3^). The rats were placed in wire mesh cages (20 × 10 × 12 cm^3^) that were mounted on a piezoelectric accelerometer (custom-made at the University of Tübingen). All movements of the rats resulted in voltage output changes, which were amplified, digitized and analyzed by a computer. The whole body acoustic startle response (ASR) amplitude was calculated as the peak-to-peak voltage outputs of the accelerometer. A loudspeaker located 40 cm away from the test cages presented the startle stimuli and a continuous white background noise (52 dB SPL). The presentation of the acoustic startle stimuli was controlled by a computer and a DA-interface (Hortmann universal function synthesizer, Hortmann, Neckartenzlingen, Germany). The startle stimuli were 20 ms tone pulses (10 kHz, 100 dB SPL) including 0.4 ms rise and fall times, which were presented with a fixed interstimulus interval of 30 s. At the beginning of the test, the rats were allowed 5 min time to acclimatize to the test chamber. Then 10 initial startle trials were presented to the animals to induce a stable baseline of ASR magnitude. Afterwards the animals were exposed to 20 startle stimuli. Half of these stimuli were presented 3.2 s after the onset of the light CS (light-tone trials), whereas the other half were presented in the absence of the CS (tone alone trials). The two trial types were presented in a pseudo-randomized manner. The difference between the tone alone and the light-tone trials was used as an operational measure of fear (e.g., Davis et al., 1993; Fendt and Fanselow, 1999). The fear potentiation was calculated as the mean relative increase in startle amplitude by the CS presentation compared to the tone-alone trials. Therefore, we set the mean startle amplitude during the tone-alone trials to 100% and calculated the relative change of the mean startle amplitude during the light-tone trials. This fear potentiation was calculated for each animal individually and then averaged for the respective groups (see also methods in Walker et al., 2009). The fear potentiated startle test was performed 24 h after the fear conditioning training.

### Histology

**Figure 2:**
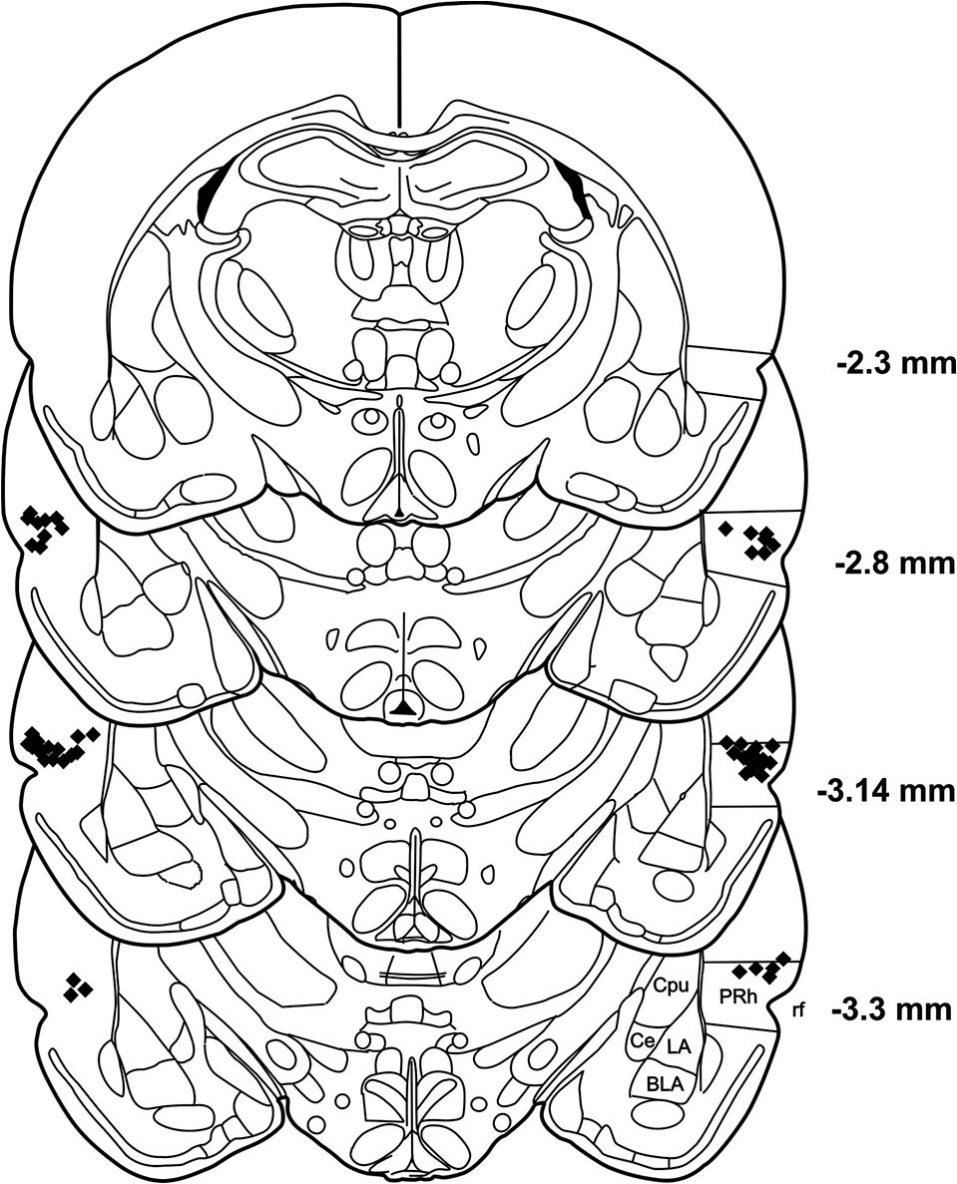
**Histological reconstruction of coronal sections of the rat brain showing the injection sites in the PRh**. BLA: basolateral amygdaloid nucleus, Ce: central amygdaloid nucleus, Cpu: caudate putamen, LA: lateral amygdaloid nucleus, PRh: perirhinal cortex, rf: rhinal fissure.

After the behavioral tests were finished, the rats were killed with an overdose of pentobarbital. The brains were removed and fixed with 8% paraformaldehyde in PBS with 20% sucrose. Coronal sections (60 μm) were taken using a freezing microtome and nissel stained. Correct placements of cannulas were verified by analyzing the stained brain slices under a light microscope. The positions of the cannulas are depicted in Figure 2.

### BDNF protein quantification

A separate group of 28 animals were fear conditioned with the identical fear conditioning protocol as used for the fear potentiated startle experiments (see above). As control a group of animals received a pseudo conditioning, i.e., an identical number of light stimuli and foot shocks that were presented in an unpaired manner to these animals. For each time point we used a group of four animals. At different time points after the fear conditioning (0, 30, 60, 90, 120 and 180 min) the animals were sacrificed and the removed brains were dissected similar to the rapid brain dissection method described by (Heffner et al., 1980). After the PRh was excised it was immediately shock-frozen with liquid nitrogen and afterwards stored at −80°C till further processing. The amount of BDNF protein in the samples was determined by using the Quantikine BDNF ELISA-kit (R&D-Systems, Wiesbaden, Germany). The tissue samples were processed according to the kit instructions.

### Statistical analysis

All data were analyzed either by using analysis of variance (ANOVA) tests followed by planned post-hoc *t*-test comparisons (alpha set at 0.05). All calculations were performed by using JMP (SAS Institute Inc., Version 8) software. A *p*-value < 0.05 was set as level of significance.

## Results

### BDNF expression in the perirhinal cortex after fear conditioning

Quantification of BDNF levels in the PRh at different time points after fear conditioning by means of BDNF-ELISA revealed a strong increase in BDNF protein at 120 min after fear conditioning training (compare Figure 1). This observation is supported by an ANOVA showing a significant effect of BDNF expression between the different time points (*F*_5,27_ = 2.8; *p* = 0.04). Post-hoc Tukey comparisons revealed significant differences between BDNF levels at 120 min and all other time points, except for the 90 min time point. However, the increase in BDNF protein at 90 min is only due to a very high amount of BDNF protein measured in the PRh of one animal, whereas all other samples were in a similar range (i.e., around 800 pg/mg) as those measured at other time points (i.e., 0–60 min). As a control, we analyzed the amount of BDNF protein in the PRh of pseudoconditioned animals at the time of the maximum BDNF proteinexpression, i.e., 120 min after fear conditioning. Here we observed no altered BDNF expression compared to time point 0 in the fear conditioned group (*p* = 0.87) but a significant difference to time point 120 min in the fear conditioned group (*p* = 0.003, Bonferroni-adapted *t*-test comparisons). Thus, the observed increase in BDNF protein 120 min after fear conditioning seems to be specific for fear learning.

### Acute interference with BDNF-TrkB-signaling in the perirhinal cortex

**Figure 3:**
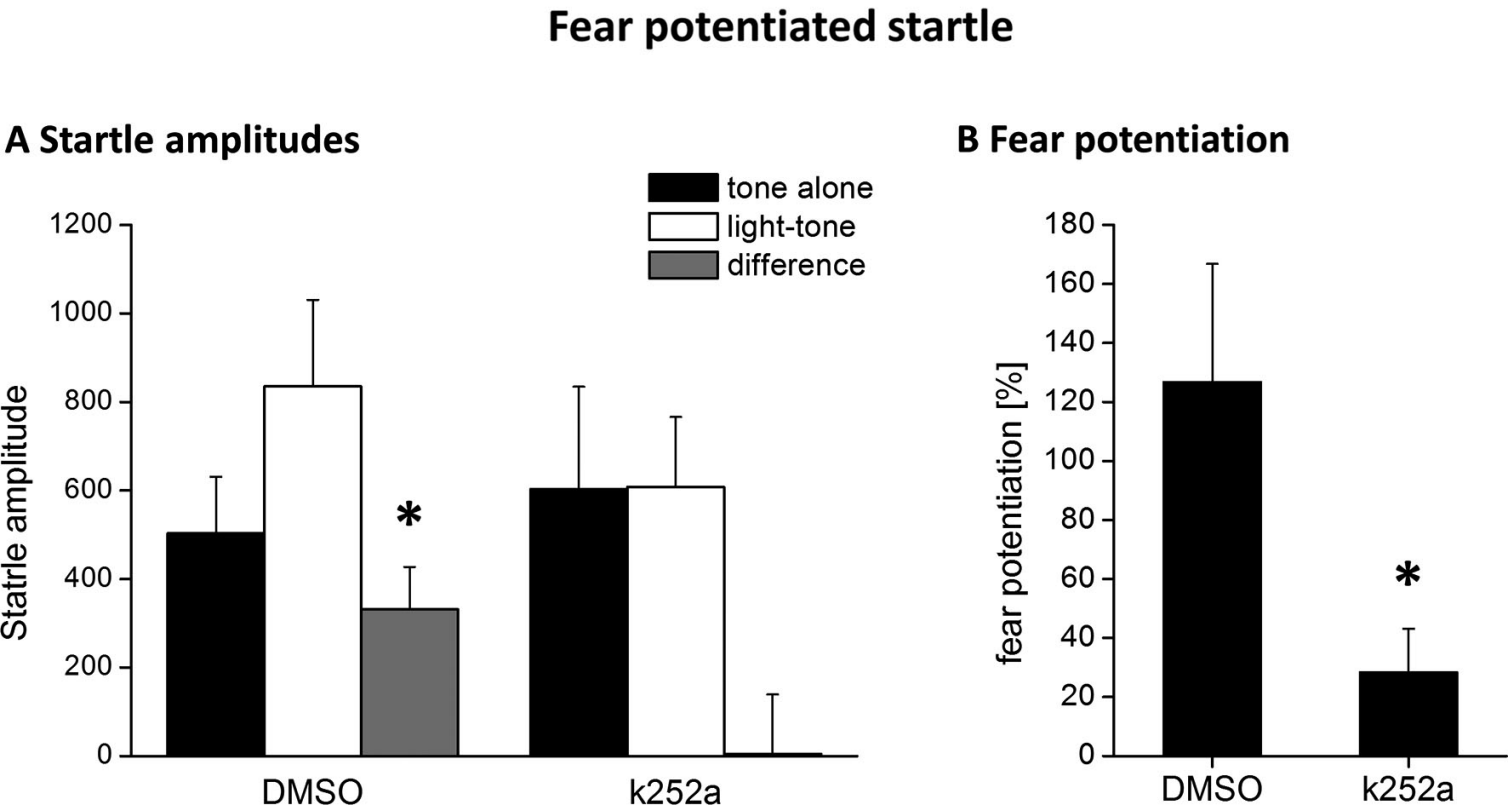
**Blocking Trk activity 120 min after fear conditioning by local k252a application impairs fear potentiation of the acoustic startle response 24 h after fear conditioning training. (A)** Mean ASR amplitude after tone-alone and light-tone trials, as well the difference scores (+SEM). **(B)** Relative fear potentiation of the startle amplitude by the presentation of the CS. * Represents a *p*-value < 0.05.

According to the known pharmacokinetic properties of k252a after injection into the brain in vivo (compare e.g., Ou et al., 2010), the injection of the tyrosine kinase inhibitor k252a into the PRh 90 min after fear conditioning was exactly matched to coincide with the maximum BDNF protein expression in this area (compare Figure 1). We observed a complete inhibition of the formation of fear memory, as indicated by a complete lack of fear potentiated startle (Figures 3A, B). We analyzed the changes in the startle amplitudes (Figure 3A) by an ANOVA using the factor “treatment” (DMSO vs. k252a) as between subject factor and the factor “phase” (tone alone vs. light-tone) as within subject factor and the interaction of these two factors. This ANOVA revealed a significant effect of the factor “phase” (*F*_1,17_ = 4.95, *p* = 0.039) but no general treatment effect (*F*_1,17_ = 0.15, *p* = 0.69). But most importantly there was a significant interaction between these two factors (phase × treatment: *F*_1,17_ = 4.71, *p* = 0.04). Post-hoc Bonferroni-adapted *t*-test comparisons revealed a significant increase in startle amplitude upon presentation of the CS in DMSO treated animals (*p* = 0.02), whereas there was no significant increase in k252a treated animals. Furthermore, it did not reveal significant differences between k252a and DMSO treated animals within the tone or light-tone trials. This lack of a significant effect in the light-tone trials might be due to high inter-individual difference in absolute startle amplitudes. In order to get rid of possible effects of inter-individual differences in the absolute startle amplitudes, we also calculated the fear potentiation of the startle amplitude upon CS presentation (Figure 3B). Here we observed a significant higher fear potentiation in DMSO treated animals compared to k252a treated animals (*p* = 0.04, *t*-test comparison).

## Discussion

In the present study, we demonstrate a learning-induced specific increase of BDNF protein in the PRh 120 min after cued fear conditioning. Furthermore, interfering with BDNF signaling in the PRh during this time window impairs the formation of fear memories, not excluding that other kinases, which are also inhibited by k252a might contribute to this effect.

By analyzing the levels of BDNF protein at different time points after fear conditioning training, we observed a strong increase of BDNF 120 min after fear conditioning took place. Since we observed this increase at 120 min only in the fear conditioned but not in the unpaired control animals, it can be ruled out that this increase of BDNF is due to the shock administration itself. A specific increase of BDNF protein upon cued fear learning has previously been demonstrated for the amygdala (Ou and Gean, 2006; Ou et al., 2010): here the BDNF protein increases 1 and 12 h after fear conditioning training. In addition, these authors showed that interfering with BDNF-TrkB-signaling at these time points prevent the consolidation of fear memory. Also for other learning tasks, like inhibitory avoidance learning, an increase in BDNF protein at distinct time points after the learning task has been described (Bekinschtein et al., 2007). Interestingly, the expression profiles of BDNF in the PRh and the amygdala seem to follow different time courses during fear learning, suggesting that BDNF-dependent consolidation processes in fear learning are not a unique and stereotypic process across different brain areas. The ELISA cannot distinguish between the pro and mature forms of BDNF. It has been shown that the processing from pro to mature BDNF is required for the induction and maintenance of LTP (e.g., Pang et al., 2004) as well as for cued and contextual fear learning (Barnes and Thomas, 2008; Wetsel et al., 2013). Interestingly both isoforms seem to have opposing effects on fear memory in that way that increased levels of mature BDNF seem to be required for the consolidation of fear memories, whereas increased levels of pro-BDNF seem to facilitate the consolidation of fear extinction (Barnes and Thomas, 2008). Given that mature BDNF has been described to be the by far dominant type of BDNF in intact mouse tissue (Matsumoto et al., 2008), it seems likely that mature BDNF is also predominantly detected and observed to be increased in our experiments.

By interfering with tyrosine kinase activity during the time of increased BDNF expression, by local application of the Trk-inhibitor k252a, we observed a complete lack of fear memory measured as fear potentiated startle. Although k252a is an unspecific tyrosine kinase inhibitor, under conditions used in our experiments it is a widely accepted tool to analyze the effects of BDNF-TrkB-signaling in vivo (compare Ou and Gean, 2006; Ou et al., 2010; Li et al., 2011; Suzuki et al., 2012; Koike et al., 2013). Given, in addition, the strong increase of BDNF-protein during this identical time window, the observed deficit in fear memory consolidation might be due to a hampered BDNF-signaling via TrkB receptors. But to finally proof the involvement of TrkB signaling in fear memory consolidation, further studies are needed that analyze either the phosphorylation of the TrkB receptors or apply the more specific BDNF scavenger TrkB-Fc into the PRh during the critical time window after fear conditioning. Nevertheless, our results provide further evidence, that PRh is not only involved in the acquisition and/or expression (Campeau and Davis, 1995; Otto et al., 2000; Schulz et al., 2004) but also in the consolidation of cued fear memories as previously described by (Sacchetti et al., 1999). Furthermore, according to our present data, BDNF seems to play an important role in the PRh enabling proper consolidation of cued fear memories.

It should be noted, that in relation to the used experimental paradigms different contributions of the PRh in fear learning have been described. So it has been shown that lesions of the PRh disrupted fear conditioning to complex, discontinuous tones or ultrasonic vocalization (e.g., 22 kHz calls) but not to simple, continuous tone stimuli (Lindquist et al., 2004; Furtak et al., 2007; Kholodar-Smith et al., 2008; Bang and Brown, 2009), suggesting that one of the mnemonic PRh functions in fear learning might be the stimulus unitization of complex auditory stimuli (Kent and Brown, 2012). However, in contrast to simple tone stimuli other simple stimuli of other sensory modalities (i.e., a light or an odor) seem to relay on proper PRh function (Campeau and Davis, 1995; Otto et al., 2000; Schulz et al., 2004). Interestingly, all studies using light stimuli as a CS measured fear learning by startle potentiation (Campeau and Davis, 1995; Schulz et al., 2004), whereas studies using acoustic stimuli as CS applied conditioned freezing (e.g., Bang and Brown, 2009). Thus it might be possible that the contribution of the PRh in fear learning could differ between different experimental approaches for assessing fear learning (i.e., conditioned freezing and fear potentiated startle). On the other hand the PRh has been shown to be required to learn a simple odor stimulus as CS in a conditioned freezing paradigm (Herzog and Otto, 1997, 1998). However, to finally resolve this issue, further experiments are required that should analyze whether the PRh is also required for conditioned freezing in response to a conditioned light stimulus. Furthermore, also the functional role of the PRh in the fear memory circuitry remains an open question. So the PRh could serve as a multimodal relay station for sensory inputs to the lateral amygdala, or it serves as an associative brain area that is directly involved in forming the CS-US association (compare also discussions in Campeau and Davis, 1995; Otto et al., 2000).

In conclusion, the present study further emphasizes the important role of PRh in the consolidation of cued fear memories. We could clearly demonstrate that fear conditioning induces a strong and specific increase in BDNF protein 120 min after fear conditioning. Furthermore, if we interfered with Trk-signaling during this critical time window, the formation of fear memories was completely blocked, as indicated by a lack of fear potentiated startle, thus suggesting an important function of BDNF in the PRh during the consolidation of fear memories.

## Author contribution

Brigitte Schulz-Klaus and Thomas Endres designed the study and performed the experiments. The manuscript was written by Thomas Endres and Brigitte Schulz-Klaus, with assistance of Volkmar Lessmann.

## Conflict of interest statement

The authors declare that the research was conducted in the absence of any commercial or financial relationships that could be construed as a potential conflict of interest.
